# Orientation-Modulated Hyperuniformity in Frustrated Vicsek–Kuramoto Systems

**DOI:** 10.3390/e28010126

**Published:** 2026-01-21

**Authors:** Yichen Lu, Tong Zhu, Yingshan Guo, Yunyun Li, Zhigang Zheng

**Affiliations:** 1 Institute of Systems Science, Huaqiao University, Xiamen 361021, China; yichenlu@stu.hqu.edu.cn (Y.L.); tongzhu@stu.hqu.edu.cn (T.Z.); ysguo@stu.hqu.edu.cn (Y.G.); 2 School of Mathematical Sciences, Huaqiao University, Quanzhou 362021, China; 3 College of Information Science and Technology, Huaqiao University, Xiamen 361021, China; 4 College of Mechanical Engineering and Automation Science and Technology, Huaqiao University, Xiamen 361021, China; 5 MOE Key Laboratory of Advanced Micro-Structured Materials, School of Physics Science and Engineering, Tongji University, Shanghai 200092, China; yunyunli@tongji.edu.cn

**Keywords:** hyperuniformity, active matter, complex systems, frustration

## Abstract

In the study of disordered hyperuniformity, which bridges ordered and disordered states and has broad implications in physics and biology, active matter systems offer a rich platform for spontaneous pattern formation. This work investigates frustrated Vicsek–Kuramoto systems, where frustration induces complex collective behaviors, to explore how hyperuniform states arise. We numerically analyze the phase diagram via the structure factor S(q) and the density variance $\langle \delta \rho^{2}\left(R\right)\rangle$. Results show that recessive lattice states exhibit Class I hyperuniformity under high coupling strength and intermediate frustration, emerging from the interplay of frustration-induced periodicity and active motion, characterized by dynamic, drifting rotation centers rather than static order. Notably, global hyperuniformity emerges from the spatial complementarity of orientation subgroups that are individually non-hyperuniform, a phenomenon termed “orientation-modulated hyperuniformity”. This work establishes frustration as a novel mechanism for generating hyperuniform states in active matter, highlighting how anisotropic interactions can yield global order from disordered components, with potential relevance to biological systems and material science.

## 1. Introduction

Disorder hyperuniformity is an exotic state of matter that bridges the gap between order (e.g., crystals and quasicrystals) and disorder (e.g., gases and liquids) states, characterized by anomalously suppressed density fluctuations at large length scales [1,2,3]. In recent years, the concept of hyperuniformity has garnered significant attention across various disciplines, including physics, materials science, and biology, such as vegetation patterns in drylands [4], leaf vein networks [5], avian photoreceptor arrangements [6], jammed disordered sphere packings [7], and even the large-scale structure of glass-like universe [8]. This unique property endows hyperuniform materials with novel physical characteristics, including isotropic photonic band gaps [9,10], enhanced mechanical properties [11], and optimal transport behavior [12,13,14,15].

Active matter systems, composed of self-driven units that consume energy to generate motion, exhibit a wide variety of collective behaviors, including flocking, swarming, and pattern formation [16,17]. Hyperuniform states have been observed in various active matter contexts, such as self-propelled particles [18,19,20,21,22,23,24,25,26], active fluids [27,28,29,30,31,32], active topological defects [33,34], DNA droplet [35], complex plasmas [36], and robotic swarms [37,38]. These findings suggest that active matter systems can spontaneously organize into hyperuniform configurations, which have implications for material design and understanding biological processes.

In this work, we continue our previous study on frustrated Vicsek–Kuramoto systems [39], which was inspired by the Kuramoto–Sakaguchi model for frustrated oscillators [40]. Frustration, arising from competing interactions or external constraints that cannot be simultaneously satisfied, is a common phenomenon in many physical systems, leading to complex energy landscapes and rich phase behaviors [41,42,43,44]. A prominent example of active matter with hydrodynamic interactions is the system of microfluidic rotors studied by Uchida and Golestanian [45]. In their work, a built-in geometric frustration, quantified by the force angle, dictates whether the rotors achieve global synchronization, enter a disordered state, or form self-proliferating spiral waves. This demonstrates how frustration can serve as a powerful control parameter in active systems. By introducing such physical mechanisms, we uncover a rich array of collective states, and the lattice state exhibits dominance for certain parameter ranges. Remarkably, we find that the recessive lattice states can exhibit hyperuniformity, characterized by suppressed long-wavelength density fluctuations. This hyperuniformity arises from the interplay between frustration-induced spatial periodicity and active motion, leading to a unique “orientation-modulated hyperuniformity” where disordered subunits assemble into a globally ordered structure. This finding extends the understanding of hyperuniformity in active-matter systems and highlights frustration as a novel mechanism for achieving hyperuniform states.

## 2. The Model and Order Metrics

Particles are described by position $r_{i}=\left(x_{i},y_{i}\right)$ and orientations $\theta_{i}$, evolving as follows:

(1a) $$\begin{array}{cc} {\dot{r}}_{i} & =vp\left(\theta_{i}\right)\;, \end{array}$$

(1b) $$\begin{array}{cc} {\dot{\theta }}_{i} & =\frac{K}{\left|A_{i}\right|}\sum_{j\in A_{i}}\left[\sin\left(\theta_{j}-\theta_{i}+\alpha \right)-\sin\alpha \right]\;, \end{array}$$

for i=1,2,…,N, where *N* is the population size, *v* is self-propulsion speed, $p\left(\theta \right)=\left(\cos\theta ,\sin\theta \right)$ is the unit vector in the orientation θ, $A_{i}\left(t\right)=\left\{j:\left|r_{i}\left(t\right)-r_{j}\left(t\right)\right|⩽d_{0}\right\}$ is the set of neighbors within coupling radius $d_{0}$, $K\left(⩾0\right)$ is coupling strength, and α is frustration. The term −sinα ensures synchronization ($\theta_{j}=\theta_{i}$) remains an equilibrium [46]. For α=0, Equation (1) reduces to the noiseless Vicsek model [47], while for v=0, it becomes the Kuramoto–Sakaguchi model [40,46].

To quantify hyperuniformity, we compute the structure factor

(2) $$S\left(q\right)=\frac{1}{N}\sum_{i,j=1}^{N}e^{iq\cdot \left(r_{i}-r_{j}\right)}\;,$$

where q is the wavevector. The structure factor provides a powerful Fourier-space characterization of the system’s density fluctuations. For a completely random Poisson point pattern, S(q)=1 for all *q*, where q≡|q| is the wavenumber. In contrast, a hallmark of hyperuniform systems is the suppression of large-scale density fluctuations, which manifests as follows:

(3) $$\lim_{q\to 0}S\left(q\right)=0.$$

Often, this approach to zero follows a power-law scaling

(4) $$S\left(q\to 0\right)∼q^{\beta },$$

with β>0, where the exponent β characterizes the degree of hyperuniformity.

We also analyze density fluctuations directly in real space, which is defined as

(5) $$\langle \delta \rho^{2}\left(R\right)\rangle =\langle {\left[\rho \left(R\right)-\langle \rho \left(R\right)\rangle \right]}^{2}\rangle \;,$$

where $\rho \left(R\right)$ is the number density within a window of radius *R*, and 〈·〉 denotes an average over different window locations. The variance generally scales as

(6) $$\langle \delta \rho^{2}\left(R\to \infty \right)\rangle ∼R^{-\lambda }\;.$$

A Poisson point configuration in *d* dimensions exhibits λ=d, while hyperuniform systems are characterized by a faster decay with λ>d. Based on asymptotic analysis at large scales, the exponent β is related to λ, and hyperuniform systems in *d* dimensions can be classified as follows:

(7) $$\langle \delta \rho^{2}\left(R\to \infty \right)\rangle ∼\left\{\begin{array}{cc} R^{-d-1},\;\beta >1 & \left(\mathrm{CLASS}\;I\right)\;,\\ R^{-d-1}\ln R,\;\beta =1 & \left(\mathrm{CLASS}\;\mathrm{II}\right)\;,\\ R^{-d-\beta },\;0<\beta <1 & \left(\mathrm{CLASS}\;\mathrm{III}\right)\;. \end{array}\right.$$

We conducted numerical simulations to investigate the performance and characteristics of our system under various conditions. For simplicity, we assume that particles are initially distributed uniformly in a two-dimensional L×L square with periodic boundary conditions. All the numerical simulations of Equation (1) were run on Python 3.12 using Euler integration with time step Δt=0.005. Each data point of order parameters was collected by averaging the last 500 time steps and different initial conditions after running 10,000 time steps of the simulation to discard the transients and reach steady states. To estimate the behavior of *S* in zero, we calculated $S(q_{n})$ for allowed wavevectors in physics $q_{n}=\left(2\pi n_{1}/L,\ldots ,2\pi n_{d}/L\right)$, with $n\in {\left(Z^{*}\right)}^{d}$ [48,49].

## 3. Results

### 3.1. Phase Diagram and Hyperuniformity

**Figure 1:**
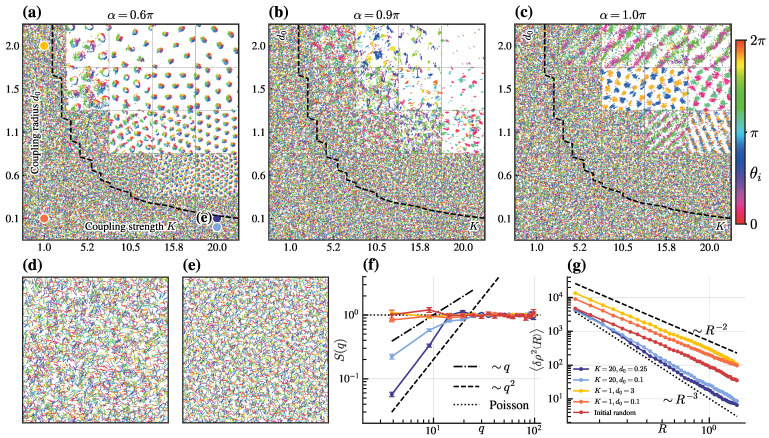
(**a**–**c**) Snapshots of $\left(K,d_{0}\right)$ phase diagram at different frustration α, with black dashed lines marking boundaries between dominant and recessive lattice states, determined by Equation (8). Arrow orientation/color indicates instantaneous direction $\theta_{i}$. (**d**,**e**) Random uniform initial state (**d**) vs. hyperuniform final state (**e**) for $K=20,d_{0}=0.25,\alpha =0.6\pi$. (**f**,**g**) Hyperuniformity of recessive states shown via structure factor S(q) in (**f**) and density variance $\langle \delta \rho^{2}\left(R\right)\rangle$ in (**g**), with black dashed lines indicating scaling law. Colored lines in (**f**) match legend in (**g**). Colored circles and label in (**a**) mark the $\left(K,d_{0}\right)$ location analyzed in (**e**–**g**). Other parameters: v=3,L=7,N=5000.

We first map the phase diagram to the $\left(K,d_{0}\right)$ plane for various α (Figure 1a–c). As α increases from 0.6π (vortex lattice state) to 0.8π (dual-cluster lattice state) and then to π (dual-lane lattice state), the system experiences transitions through distinct lattice configurations, dominating at high coupling strengths and radius, while recessive lattice states emerge at relatively low *K* and $d_{0}$, which have lost their stability according to the linear stability analysis of the disordered uniform state, while still maintaining macroscopic spatial uniformity. In our previous work [39], these states were defined as dominant and recessive lattice states, respectively, by the critical boundary

(8) $$\frac{2\pi }{k^{*}\left(d_{0}\right)}=\frac{2v}{K}\;,$$

where $k^{*}\left(d_{0}\right)$ is the most unstable wavevector from linear stability analysis of the disorder uniform state. Linear stability analysis indicates that for |α|>π/2, the disordered uniform state becomes unstable via a Hopf–Turing bifurcation, demonstrating that recessive lattice states are not simply disordered despite their appearance. The emergence of recessiveness arises non-trivially from the interplay between frustration-induced pattern formation and active motion, where the lattice constant—the mean spacing between neighboring unit cells, $2\pi /k^{*}\left(d_{0}\right)$—is too short to accommodate the effective diameter of a unit cell, given by 2v/K. This forces cells to overlap, leading to macroscopically uniform patterns while preserving an underlying tendency toward periodic order, ultimately giving rise to ordered configurations.

Notably, the recessive lattice state with high *K* and low $d_{0}$ exhibits hyperuniformity. This state emerges from random initial conditions (Figure 1d, red lines in Figure 1f,g), forming a hyperuniform configuration (Figure 1e, dark blue line in Figure 1f,g). The structure factor S(q) and the density variance $\langle \delta \rho^{2}\left(R\right)\rangle$ confirm hyperuniform scaling, with

(9) $$\begin{array}{c} S\left(q\to 0\right)∼q^{\beta },\beta >1\;,\\ \langle \delta \rho^{2}\left(R\to \infty \right)\rangle ∼R^{-\lambda },\lambda >2\;, \end{array}$$

(dark and light blue lines in Figure 1f,g), corresponding to class I hyperuniformity as seen in crystals and quasicrystals. In contrast, for low *K* and either high or low $d_{0}$, the recessive lattice state becomes disordered and loses hyperuniformity. This character is signaled by

(10) $$\begin{array}{c} S\left(q\right)\approx 1\;,\\ \langle \delta \rho^{2}\left(R\to \infty \right)\rangle ∼R^{-2}\;, \end{array}$$

as indicated by the orange and yellow lines in Figure 1f,g.

According to Equation (1b), coupling strength *K* affects the angular velocity of particles, which in turn determines the cell diameter. When *K* is sufficiently large, particles within a unit cell are rapidly rotating and densely packed, strongly suppressing long-wavelength density fluctuations and thereby producing hyperuniformity. However, at low *K*, particles within each unit cell are loosely packed, allowing significant density fluctuations at long wavelengths, thus losing hyperuniformity. Therefore, strong local alignment is crucial for achieving hyperuniformity in this system.

**Figure 2:**
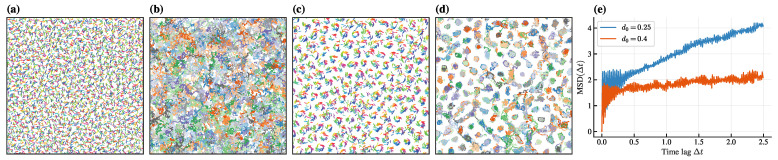
Snapshots and trajectories of rotation centers in (**a**,**b**) hyperuniform state ($d_{0}=0.25$) and (**c**,**d**) dominant lattice state ($d_{0}=0.4$). Only 25% of the particle trajectories are shown in (**b**,**d**) for clarity. (**e**) MSD of rotation centers for hyperuniform state (blue) and dominant lattice state (orange) after initial transient ($t_{0}=2.5$). Other parameters: K=24,α=0.6π,v=3,L=7,N=5000.

It is worth noting that the states observed earlier are active hyperuniform states, not dominant lattice states with extremely short periodicity. This is confirmed by analyzing the trajectories of rotation centers, defined as

(11) $$c_{i}\left(t\right)=r_{i}\left(t\right)-\frac{v}{{\dot{\theta }}_{i}\left(t\right)}\left[\begin{array}{c} \sin\theta_{i}\left(t\right)\\ -\cos\theta_{i}\left(t\right) \end{array}\right]\;,$$

where $c_{i}$ removes the circular motion of $r_{i}$ for clearer visualization. In the hyperuniform state (Figure 2a,b), rotation centers drift disorderly throughout space, while in the dominant lattice state (Figure 2c,d), they remain confined near fixed lattice points. For further quantification, we compute the mean squared displacement (MSD) of rotation centers:

(12) $$\mathrm{MSD}\left(t\right)=\langle {\left|c_{i}\left(\Delta t+t_{0}\right)-c_{i}\left(t_{0}\right)\right|}^{2}\rangle \;,$$

where 〈·〉 averages over all particles. As shown in Figure 2e, the hyperuniform state exhibits sustained growth of MSD over time, indicating diffusive behavior, while the dominant lattice state shows saturation of MSD, indicating localization. Thus, the hyperuniform state is dynamic, with suppressed long-wavelength density fluctuations. The animations of these two states are further illustrated in Supplementary Videos S1 and S2.

**Figure 3:**
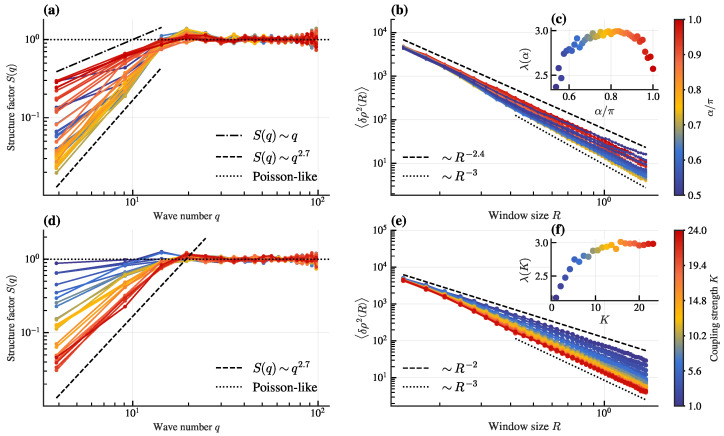
Structure factor and density fluctuations scaling across varying control parameters. (**a**) Structure factor S(q) and (**b**) density variance $\langle \delta \rho^{2}\left(R\right)\rangle$ scaling at varying frustration α (K=24). Dashed lines show reference scalings for Poisson-like and hyperuniform behavior. Inset (**c**): Scaling exponent of (**b**) from Equation (6) vs. α. Different colors in (**a**–**c**) correspond to α values indicated in color bar at right. (**d**,**e**) Analogous plots to (**a**,**b**), showing the evolution of S(q) and $\langle \delta \rho^{2}\left(R\right)\rangle$ scaling with increasing coupling strength *K* (α=0.8π). Inset (**f**): Corresponding scaling exponent of (**e**) vs. *K*. Different colors in (**d**–**f**) correspond to *K* values indicated in color bar at right. Other parameters: $d_{0}=0.25,v=3,L=7,N=5000$.

To study how hyperuniformity changes with parameters α and *K*, we analyze S(q) and $\langle \delta \rho^{2}\left(R\right)\rangle$ for fixed $d_{0}=0.25$ (Figure 3). Increasing α from 0.5π to π at fixed K=24 transitions the system from Class III to Class I and back to Class III hyperuniformity, with the exponent λ (inset of Figure 3c) varying non-monotonically and reaching its maximum value over a broad range centered around α=0.8π. Besides, increasing *K* from 1 to 24 (truncated at 24 since the system enters the dominant lattice state beyond this) at fixed α=0.8π drives a transition from non-hyperuniform (Poisson-like configuration) to Class I hyperuniform. Inset of Figure 3f displays the scaling law extracted from the density variance, evidencing a clear trend toward stronger suppression of long-wavelength fluctuations with increasing coupling strength.

The non-monotonic variation of the hyperuniformity class with α (Figure 3c) suggests an optimal range of frustration for achieving the most ordered (Class I) hyperuniform states. This implies that pronounced hyperuniformity persists over a broad range of frustration, rather than being restricted to a finely tuned value. Similarly, the transition from non-hyperuniform to Class I behavior with increasing *K* (Figure 3f) underscores that strong local alignment is crucial for suppressing long-wavelength density fluctuations effectively.

**Figure 4:**
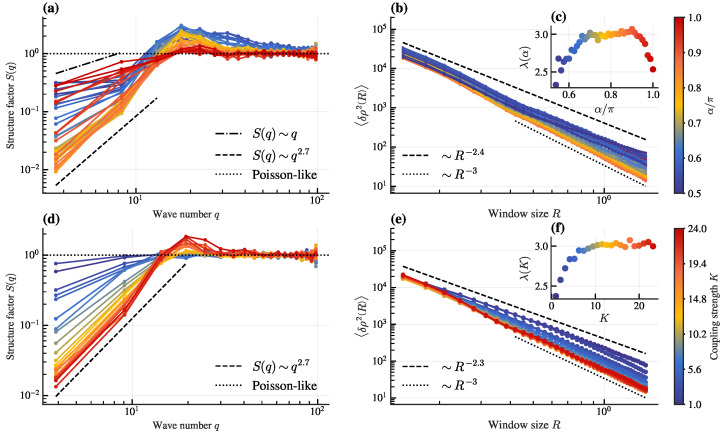
Structure factor and density-fluctuation scaling for a larger system size (*N* = 20,000). The panels (**a**–**c**) and (**d**–**f**) correspond to varying frustration α (at fixed K=24) and coupling strength *K* (at fixed α=0.8π), respectively, analogous to Figure 3. Other parameters are the same as in Figure 3.

To ensure that the observed hyperuniformity is not a finite-size artifact, we perform simulations with a larger system size (*N* = 20,000) while keeping other parameters consistent (Figure 4). The results confirm that both S(q) and $\langle \delta \rho^{2}\left(R\right)\rangle$ maintain their hyperuniform scaling behavior, demonstrating the robustness of hyperuniformity in our system.

**Figure 5:**
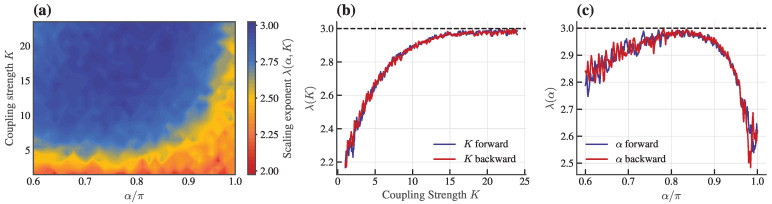
Phase diagram of hyperuniformity and hysteresis analysis. (**a**) Heat map of the scaling exponent from Equation (6) in the parameter space of coupling strength *K* and frustration α. The exponent λ (color-coded) serves as an indicator of hyperuniformity (λ<−2). (**b**,**c**) The exponent λ as a function of (**b**) *K* at fixed α=0.8π and (**c**) α at fixed K=24, adiabatically sweeping the parameters forward and backward to analyze hysteresis behavior. Other parameters are same as in Figure 3.

For a comprehensive overview of hyperuniformity across the parameter space, we construct a heat map of the scaling exponent λ from Equation (6) in the (α,K) plane (Figure 5a). This map reveals distinct behaviors: disordered states with λ≈2, Class III (2<λ<3), and Class I (λ≈3). Consistent with the description in Figure 3, strong hyperuniformity (Class I) emerges at high *K* and intermediate α, while weaker hyperuniformity (Class III) appears at lower *K* and extreme α values.

To probe the nature of behavioral evolution, we examine hysteresis by adiabatically varying *K* and α, respectively (Figure 5b,c). The scaling exponent λ did not exhibit significant hysteresis when sweeping forward and backward, indicating that these transitions between disordered and hyperuniform states are smooth, highlighting the system’s insensitivity to initial conditions and instantaneous state, thus underscoring the robustness of hyperuniformity in these regimes. This implies that the hyperuniform states are thermodynamically stable states accessible from both ordered and disordered initial conditions, enhancing their potential relevance for experimental realization, where control over initial states is often limited.

### 3.2. Orientation-Modulated Hyperuniformity

**Figure 6:**
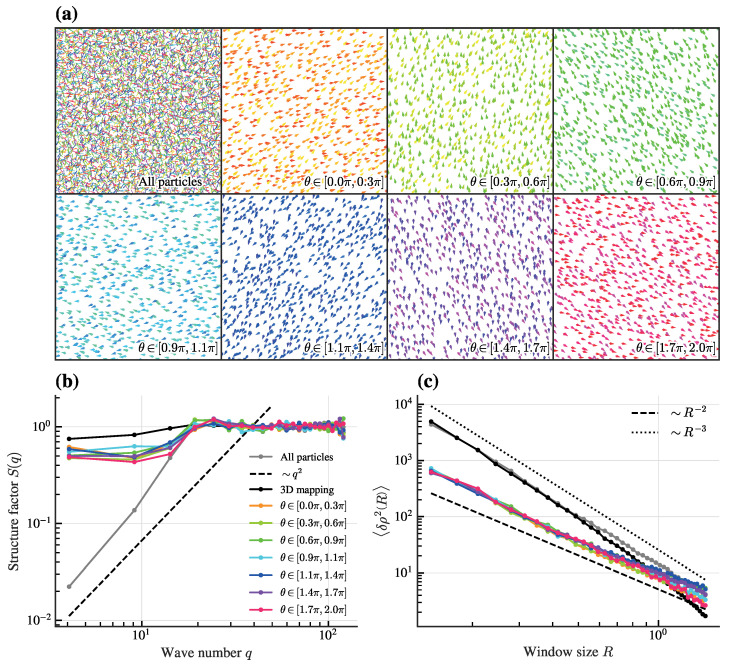
The impact of angular selectivity on hyperuniformity. Particle configurations within specific angular ranges (**a**) exhibit non-hyperuniform configurations, as quantified by the structure factor S(q) in (**b**) and density variance $\langle \delta \rho^{2}\left(R\right)\rangle$ in (**c**). Colored lines in (**c**) match legend in (**b**). Parameters: *K* = 24, $d_{0}$ = 0.25, α = 0.8π, *v* = 3, *L* = 7, *N* = 5000.

In contrast to traditional hyperuniform systems, where non-self-propelled particles are driven by external fields or isotropic interactions, our system reveals a distinct mechanism driven by angular selectivity in active matter, whose hyperuniformity here is dynamic and arises from the self-organized motion of particles with orientation-dependent interactions. To clarify how angular selectivity shapes global order, we examine particle configurations within defined orientation ranges. Particles with orientations $\theta_{i}$ uniformly distributed in [0,2π) are grouped into subsets of angular width Δθ=2π/7 divided at $\theta_{c}=n\Delta \theta$ with n=0,1,…,6. As shown in Figure 6a, particles within each bin are spatially non-hyperuniformly distributed.

We quantify the spatial hyperuniformity of these orientation-selected subsets by computing the structure S(q) and $\langle \delta \rho^{2}\left(R\right)\rangle$ for these orientation-specific subsets (Figure 6b,c). For every subset, S(q) approaches a finite constant as q→0 and $\langle \delta \rho^{2}\left(R\to \infty \right)\rangle$ scales as $R^{-2}$, confirming the absence of hyperuniformity within any single orientation bin. Nevertheless, the full system—integrating all orientations—exhibits clear hyperuniform signatures, as demonstrated earlier. This behavior is robust against changes in $\theta_{c}$ and Δθ.

Importantly, when orientations are treated as an additional dimension (mapping each particle to $\left(x_{i},y_{i},\theta_{i}\right)$ in a 3D space), the resulting 3D point set is not hyperuniform (black lines in Figure 6b,c). This confirms that hyperuniformity in our system is a projection effect: it emerges only when the orientational degree of freedom is integrated out, underscoring the anisotropic nature of the inter-particle correlations.

Unlike multi-hyperuniform systems, in which each subspecies is individually hyperuniform [6], the phenomenon here is fundamentally different: individual orientation subsets are not hyperuniform, yet their mutual spatial complementarity yields a globally hyperuniform state. This interplay defines a distinct mechanism, which we term “orientation-modulated hyperuniformity.” In this mechanism, global order does not arise from hyperuniform subsets, but from the specific spatial arrangement and mutual compensation of multiple non-hyperuniform subpopulations. Such a design principle may be especially relevant to biological groups, where individuals in distinct behavioral states can collectively produce highly ordered macroscopic patterns, even when each subgroup alone is spatially disordered. While still speculative, this mechanism could be particularly relevant in systems where individuals assume distinct roles or states based on internal or external cues. For instance, in bacterial colonies, cells in different metabolic states might spatially organize to optimize resource utilization, leading to emergent hyperuniform patterns at the colony level. Similarly, in animal groups, individuals in different behavioral states (e.g., foraging vs. resting) could spatially arrange themselves to enhance group cohesion and information transfer, resulting in hyperuniform distributions despite the disordered nature of each behavioral subgroup.

## 4. Discussion

This study demonstrates that frustrated Vicsek–Kuramoto systems can spontaneously generate disordered hyperuniform states, unveiling a novel mechanism—orientation-modulated hyperuniformity—where global order emerges from the spatial complementarity of multiple non-hyperuniform subpopulations. Unlike classical hyperuniform systems induced by external fields or isotropic interactions, hyperuniformity here arises from the intrinsic interplay between frustration-induced spatial periodicity and active motion.

The recessive lattice states identified under certain parameter regimes exhibit clear Class I hyperuniform scaling, with $S\left(q\to 0\right)∼q^{2.7}$ and $\langle \delta \rho^{2}\left(R\to \infty \right)\rangle ∼R^{-3}$ for the most hyperuniform cases, and their hyperuniform scaling behavior is robust against finite-size effects. This hyperuniformity is not static but dynamic, as confirmed by the disordered drifting of rotation centers, distinguishing it from crystalline or quasicrystalline orders. The continuous variation of the hyperuniformity class with frustration α and coupling strength *K*, together with the absence of hysteresis, suggests that these states are robust and accessible over a broad parameter range.

A key insight from our analysis is the anisotropic nature of density fluctuations. While the full system in 2D real space is hyperuniform, subsets defined by orientation bins are not. Furthermore, the 3D point set (x,y,θ) fails to be hyperuniform, indicating that hyperuniformity is a projection effect—it manifests only when orientational degrees of freedom are integrated out. This underscores that the suppression of density fluctuations is directional: effective in real space but absent in the full configuration space, including orientations. Such anisotropic correlations highlight that global order stems from the specific mutual arrangement of orientation groups, each of which is itself spatially disordered.

This mechanism differs fundamentally from multi-hyperuniform systems, where each subspecies is hyperuniform on its own. Instead, in our system, hyperuniformity is an emergent property resulting from the collective interplay of disordered subgroups. Frustration not only dictates the emergence of lattice-like patterns but also orchestrates the angular distribution of particles in a way that their spatial positions become hyperuniform upon averaging over all orientations.

Several questions remain open for future investigation. The precise role of noise, the effect of chirality and heterogeneity, and the generalization to three-dimensional active systems warrant further exploration. It would also be valuable to examine whether a similar orientation-modulated ordering occurs in other active-matter systems with non-reciprocal or vision-based interactions. Form an experimental perspective, our model could be realized in chiral active particles such as the microfluidic rotors studied by Uchida and Golestanian [45], where geometric frustration (e.g., force angle) can be tuned to induce synchronization or pattern formation—similar to our model. Similarly, vibrated granular matter (e.g., anisotropic particles on vibrating plates [50,51]) or engineered micro-swimmers (e.g., colloidal swarmalators [52] or Janus particles [53]) allow precise tracking of particle orientations via high-speed imaging.

In summary, our work establishes frustration as a potent and generic route to hyperuniformity in active matter and introduces “orientation-modulated hyperuniformity” as a distinct class of disordered hyperuniform states. It highlights how selective interactions in angular space can give rise to superior spatial order at large scales—a concept that may extend beyond active matter to other spatially organized multi-component systems.

## Data Availability

The data presented in this study are available on request from the corresponding author.

## Supplementary Materials

The following supporting information can be downloaded at: https://www.mdpi.com/article/10.3390/e28010126/s1, Video S1: Animation of particle trajectories in the hyperuniform state shown in Figure 2a; Video S2: title: Animation of particle trajectories in the dominant lattice state shown in Figure 2c.
